# Surgical treatment of intestinal perforation in Behçet Syndrome: an unusual presentation

**DOI:** 10.11604/pamj.2018.30.230.15950

**Published:** 2018-07-26

**Authors:** Carla Sofia Vicente, António Dias Freitas

**Affiliations:** 1 Centro Hospitalar Lisboa Central, Lisboa, Portugal; 2 Hospital de Cascais, Cascais, Portugal

**Keywords:** Behçet, vasculitis, intestinal perforation

## Abstract

Behçet syndrome is a chronic, recurring, systemic disorder characterized by the histopathologic finding of nonspecific vasculitis in multiple organs. Behçet syndrome involves the gastrointestinal tract in 10-50% of patients; The main sites of involvement are the terminal ileum and cecum. In patients with Behçet syndrome, CT is advocated for early detection of complications as well as for exclusion of other abdominal pathologic conditions but there is no specific exam. The report of histology in conjunction with the clinical history and the presence of oral ulcers, uveitis and suspected cutaneous lesions suggests the diagnosis. The optimal medical treatment of Behçet syndrome has not yet been well established. In rare cases surgery must be required to control the disease. The authors report one case of Behcet Syndrome presenting with intestinal perforation.

## Introduction

Behçet syndrome is a chronic, recurring, systemic disorder characterized by the histopathologic finding of non-specific vasculitis in multiple organs. The diagnosis is based primarily on clinical criteria because of the non-specificity of the histopathologic findings. Previous sets of diagnostic criteria included a long list of minor symptoms or signs and included clinical features with insufficient frequency [1]. Therefore, new diagnostic criteria that were simple and more specific and excluded rarer manifestations appeared to be necessary. As a result, the International Study Group for Behçet's Disease proposed new diagnostic criteria in 1990 [2]. These criteria require the presence of oral ulcers plus any two of the following: genital ulcers, typical eye lesions, typical skin lesions, or a positive result of a pathergy test (i.e, a sterile pustule developing after 24-48 hours at the site of a needle prick to the skin). Behçet syndrome involves the gastrointestinal tract in 10-50% of patients; such involvement results from vasculitis in the small vessels of the bowel wall, more frequently in the venules [3-5]. The main sites of involvement are the terminal ileum and cecum, but the upper gastrointestinal tract including the esophagus and, rarely, the stomach can be affected [6].

## Patient and observation

**Figure 1:**
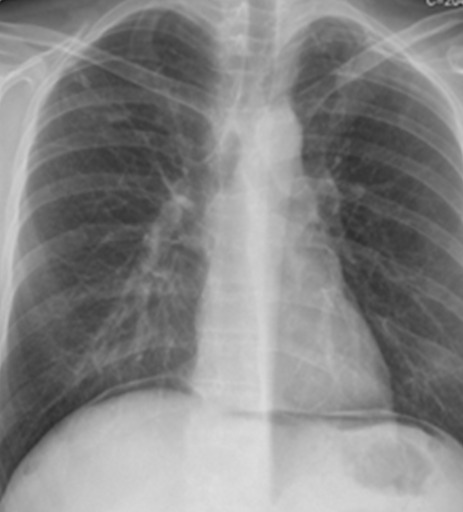
Thoracic radiography with pneumoperitoneum

**Figure 2:**
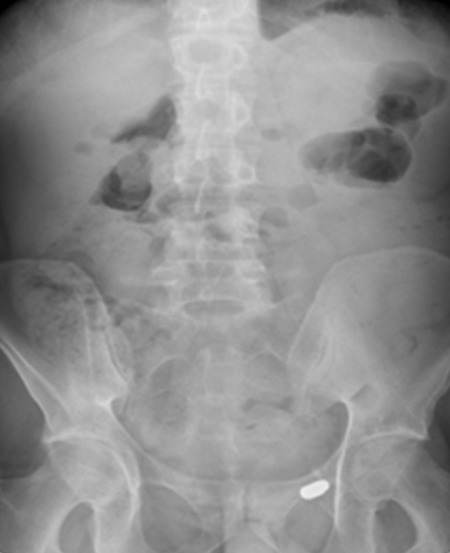
Abdominal radiography with pneumoperitoneum

**Figure 3:**
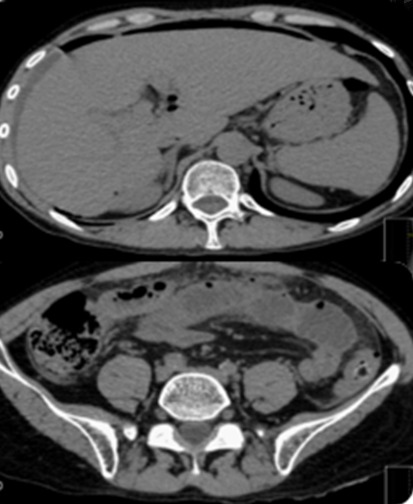
CT scan showing intra-peritoneal fluid pneumoperitoneum and bowel perforation

**Figure 4:**
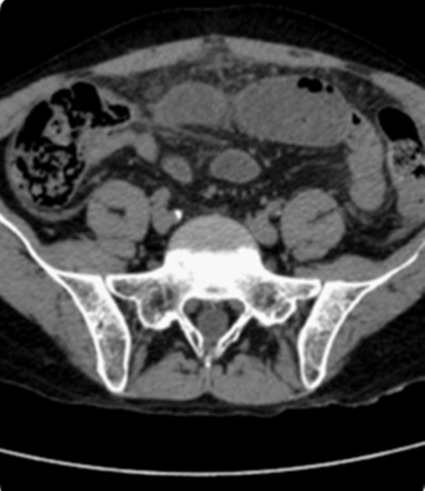
CT scan showing bowel thickening

**Figure 5:**
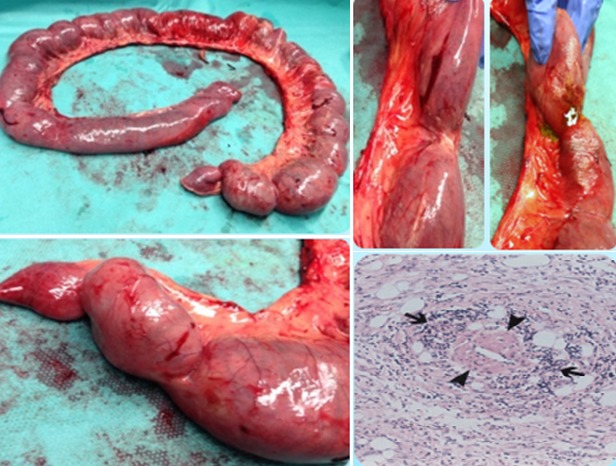
Bowel perforations at surgery

38 years-old man with personal history of drug addiction in the past, VIH1 and VHC Infection, cerebral toxoplasmosis, pulmonary tuberculosis, and uveitis in the previous year (diagnosis made by an Ophthalmologist). The patient proceeded to the emergency department with abdominal pain, and vomiting, in the previous 24h. On examination, his vital signs were stable, but he presented an acute abdomen with peritoneal rebound. He revealed also maculo-pustular lesions in the limbs. The laboratory results of the patient showed no anemia, an elevated leukocyte count and a high C-reactive protein (Hb 13.3g/dL, WBC 7300, Plat 112000. CRP 1.81mg/dl). We started the approach by asking a thoracic radiography that showed a pneumoperitoneum (Figure 1, Figure 2). This way, an urgent CT scan revealed signs of pneumoperitoneum with “intra-peritoneal fluid specially in the pelvic cavity and an extension of thickened small bowel”. In the same exam we found a small “collection of enteric fluid” (Figure 3, Figure 4). The patient underwent a laparotomy where we found a multi-segmental disease with multiples stenosis, one of which was perforated. We proceed to the resection of that segment of small bowel and a termino-terminal anastomosis. He was asymptomatic at the time of discharge from hospital. Histology revealed “small areas of mucosal necrosis, lacerations and perforations, and an important granulocytic inflammation (…) focal proliferation of capillaries which are in marked stasis. Venous congestion in mesenteric vessels. These aspects should be contextualized with the clinic, imaging and systemic study of the patient but are suggestive of Behçet's syndrome (ischemic ulcerations)” (Figure 5).

## Discussion

The presence of ulcers is the radiologic and pathologic hallmark of intestinal involvement. Two types of ulceration occur: localized and diffuse. In cases of localized lesions, which are commonly seen in the ileocecal region, the mucosal ulcers are deep, often penetrating to the serosal surface with common occurrence of perforation [6]. In contrast, diffuse lesions are commonly seen in the colon and may occur as multiple discrete, punched-out ulcers. The smaller such lesions appear as aphthous ulcers, simulating Crohn disease [7,8]. Although the exact cause of Behçet syndrome remains unknown, it has been speculated that viral infection, environmental factors, and autoimmune mechanisms might play a role [9,10]. Also, there are some associations with human leukocyte antigens in Eastern countries [11]. In contrast to Reiter disease, in which the frequency of HLA-B27 is high, HLA-B5 and HLA-B51 are associated with Behçet syndrome. Although it is impossible or very difficult to detect mucosal ulcers, computed tomography (CT) has advantages in demonstrating bowel wall thickening and lesions in the extraluminal space. Therefore, in patients with Behçet syndrome, CT is advocated for early detection of complications as well as for exclusion of other abdominal pathologic conditions [12-14]. Furthermore, CT might allow prediction of the possibility of complications [12]. There is no specific exam. The report of histology in conjunction with the clinical history and the presence of oral ulcers, uveitis and suspected cutaneous lesions suggests the diagnosis. The differential diagnosis should include all the intestinal inflammatory diseases. The optimal medical treatment of Bechet syndrome has not yet been well established. The natural history of exacerbation and remission makes evaluation of therapy difficult [4]. Corticosteroids are the mainstay of medical therapy in Behçet syndrome. Other medical treatments such as azathioprine, colchicine, dapsone, levamisole, thalidomide, and immunosuppressive therapies have been advocated [13,14]. In rare cases surgery must be required to control the disease.

## Conclusion

Patients in whom medical therapy has been unsuccessful or who have extensive disease or complications such as perforation, hemorrhage, or peritonitis should be considered for surgery, as in the present case.

## Competing interests

The authors declare no competing interests.
